# Heterologous neutralization breadth persists despite B-lymphocyte dysfunction in chronic HIV-1 infection

**DOI:** 10.1186/1742-4690-9-S2-P103

**Published:** 2012-09-13

**Authors:** MK Murphy, S Boliar, TC Tran, DG Carnathan, WS Armstrong, G Silvestri, CA Derdeyn

**Affiliations:** 1 Emory University, Atlanta, GA, USA

## Background

Of the millions globally infected with HIV-1, only 20-30% will develop broadly neutralizing antibodies. To date, no one has measured this phenomenon in a cohort of subjects for which multiple aspects of B-lymphocyte dysfunction have been evaluated in parallel.

## Methods

In 16 viremic seroconverters, the cross-clade neutralizing activity of plasma was investigated using a panel of thirteen clade A, B, and C HIV-1 envelope (Env) pseudotyped virions, which represented three tiers of sensitivity. The neutralization IC50 was calculated for each plasma-Env combination, and these data were used to determine a breadth (how many Envs were neutralized) and potency (the strength of neutralization) score for each seroconverter. Additionally, the level of plasma antibodies that bound to the monomeric form of a subtype B Env gp120 (HIV-1 BaL) was quantitated.

## Results

A range of neutralization breadth emerged: three plasma samples (19%) demonstrated widespread neutralizing activity against this panel of Envs, while five subjects (31%) exhibited a complete lack of detectable neutralization at the lowest dilution of plasma tested (1:100). No correlation was observed between neutralization breadth or potency and parameters of B-lymphocyte dysfunction (PD-1, BTLA), immune activation (Ki-67, CD95), or disease progression (CD4 T cell count, plasma viral load). The level of total IgG in each plasma sample, however, did significantly correlate with both neutralization breadth and potency. Like total IgG, anti-gp120 binding antibodies also positively correlated, but, in this case, the correlations only trended toward significance. Anti-gp120 binding antibodies did not correlate with parameters of B-lymphocyte dysfunction, immune activation, disease progression, or total IgG level.

## Conclusion

These findings demonstrate that even in chronically HIV-1-infected subjects in whom B-lymphocytes display multiple indications of dysfunction, antibodies that mediate cross-clade neutralization breadth (particularly anti-gp120 binding and other IgG antibody specificities) continue to circulate in plasma.

